# Olfactory sampling volume for pheromone capture by wing fanning of silkworm moth: a simulation-based study

**DOI:** 10.1038/s41598-024-67966-y

**Published:** 2024-08-02

**Authors:** Toshiyuki Nakata, Daigo Terutsuki, Chihiro Fukui, Tomoya Uchida, Kohei Kanzaki, Taito Koeda, Sakito Koizumi, Yuta Murayama, Ryohei Kanzaki, Hao Liu

**Affiliations:** 1 Graduate School of Engineering, Chiba University, Chiba, Japan; 2 Department of Mechanical Engineering and Robotics, Faculty of Textile Science and Technology, Shinshu University, Nagano, Japan; 3 Graduate School of Science and Engineering, Chiba University, Chiba, Japan; 4 Department of Mechano-Informatics, Graduate School of Information Science and Technology, The University of Tokyo, Tokyo, Japan; 5 Research Center for Advanced Science and Technology, The University of Tokyo, Tokyo, Japan

**Keywords:** Mechanical engineering, Biomechanics

## Abstract

Odours used by insects for foraging and mating are carried by the air. Insects induce airflows around them by flapping their wings, and the distribution of these airflows may strongly influence odour source localisation. The flightless silkworm moth, *Bombyx mori*, has been a prominent insect model for olfactory research. However, although there have been numerous studies on antenna morphology and its fluid dynamics, neurophysiology, and localisation algorithms, the airflow manipulation of the *B. mori* by fanning has not been thoroughly investigated. In this study, we performed computational fluid dynamics (CFD) analyses of flapping *B. mori* to analyse this mechanism in depth. A three-dimensional simulation using reconstructed wing kinematics was used to investigate the effects of *B. mori* fanning on locomotion and pheromone capture. The fanning of the *B. mori* was found to generate an aerodynamic force on the scale of its weight through an aerodynamic mechanism similar to that of flying insects. Our simulations further indicate that the *B. mori* guides particles from its anterior direction within the ~ 60° horizontally by wing fanning. Hence, if it detects pheromones during fanning, the pheromone can be concluded to originate from the direction the head is pointing. The anisotropy in the sampling volume enables the *B. mori* to orient to the pheromone plume direction. These results provide new insights into insect behaviour and offer design guidelines for robots for odour source localisation.

## Introduction

Insects use a myriad of volatile chemicals in the air as cues or signals during foraging and mating. Chemicals released from odour sources, such as food or pheromone glands, disperse with airflow, and their concentrations vary with time and space^1–3^. Despite the complexity of the natural environment, insects can identify the location of odour sources by sampling and detecting chemicals at their antennae and navigating based on the instantaneous concentration of chemicals^4,5^. The search for odour sources using antennae, particularly the sensitivity of antennae to odours and localisation algorithms, has attracted increasing attention^6,7^. However, odours move with airflow, and the aerodynamics in the vicinity of insects are also crucial for odour source localisation.

For decades, the silkworm moth, *Bombyx mori*, has been used as a model organism for olfactory research. *B. mori* is a domesticated insect used in silk farming through artificial selection. This domestication resulted in phenotypic changes, such as a larger body and smaller wings, rendering the silkworm flightless^8^. However, their olfactory sensitivity to pheromones remains unchanged from that of the wild^9^. Moreover, *B. mori* males exhibit walking behaviour induced by the female sex pheromone bombykol. When exposed to bombykol, *B. mori* males initiate a zigzag-walking exploratory behaviour consisting of translational and rotational movements to localise females^10,11^.

Despite losing their flight ability, *B. mori* continues to flap upon detection of sex pheromones. Many insects, such as honeybees, are known to flap their wings on the ground ventilating their nests by fanning^12,13^. Nest ventilation by bees is achieved by inducing airflow downstream, but airflow is always induced upstream as well. Hence, even without flight, wing flapping can induce airflow and can guide odour molecules towards the antenna^14,15^. The active entrainment of air by flapping wings moves the fluid closer to the antennae, enabling more fluid to be sampled and increasing the flow velocity, thus thinning the boundary layer near the antennae/sensor to render it easier to capture the pheromone^16^. Therefore, the “fanning” by *B. mori* male is effective in the search for pheromone sources^16^. Airflow entrainment by flapping wings may also be crucial in flying insects^17,18^, such as *Drosophila*. Because *B. mori* frequently changes direction during exploration, the three-dimensional direction and distance from which air is directed to their antennae are crucial for understanding their search behaviour. However, the three-dimensional volume of air sampled by wing-fanning *B. mori* males has not been measured.

The *B. mori* has long served as a model insect for olfactory research. However, compared to antenna morphology and its fluid dynamics^16^, neurophysiology^11,19^, and localisation algorithms^20–22^, the airflow manipulation of the *B. mori* by fanning has not been thoroughly investigated. In this study, we performed computational fluid dynamics (CFD) analyses of flapping *B. mori* using wing kinematics reconstructed from high-speed video. Furthermore, we investigated the aerodynamic mechanisms of force and airflow generation by flapping of *B. mori* and quantified the three-dimensional sampling volume of pheromones from the flapping *B. mori* in zero wind. These results provide further insights into the search behaviour of *B. mori* and offer new design guidelines for odour source-searching robots.

## Materials and methods

### Insects and odorants

Adult male silkworm moths, *B. mori* (a hybrid strain of Kinshu × Showa), were purchased from Ueda Sansyu, Nagano, Japan. The principal sex pheromone component of *B. mori*, bombykol ((E, Z)-10,12-hexadecadien-1-ol), was used for stimulation. Bombycol^23^ alone stimulates the full suite of odour searching behaviour^24^. The diffusion coefficient for bombykol in air (20 °C, 101.3 kPa) is 2.5 × 10^−6^ m^2^ s^−1^^25^. Using the kinematic viscosity of air under the same conditions (1.5 × 10^−5^ m^2^ s^−1^), the Schmidt number defined as the ratio of momentum diffusion and mass diffusion (kinematic visocity divided by the diffusion coefficient) is calculated to be 6.0.

### Reconstruction of wing kinematics

**Figure 1:**
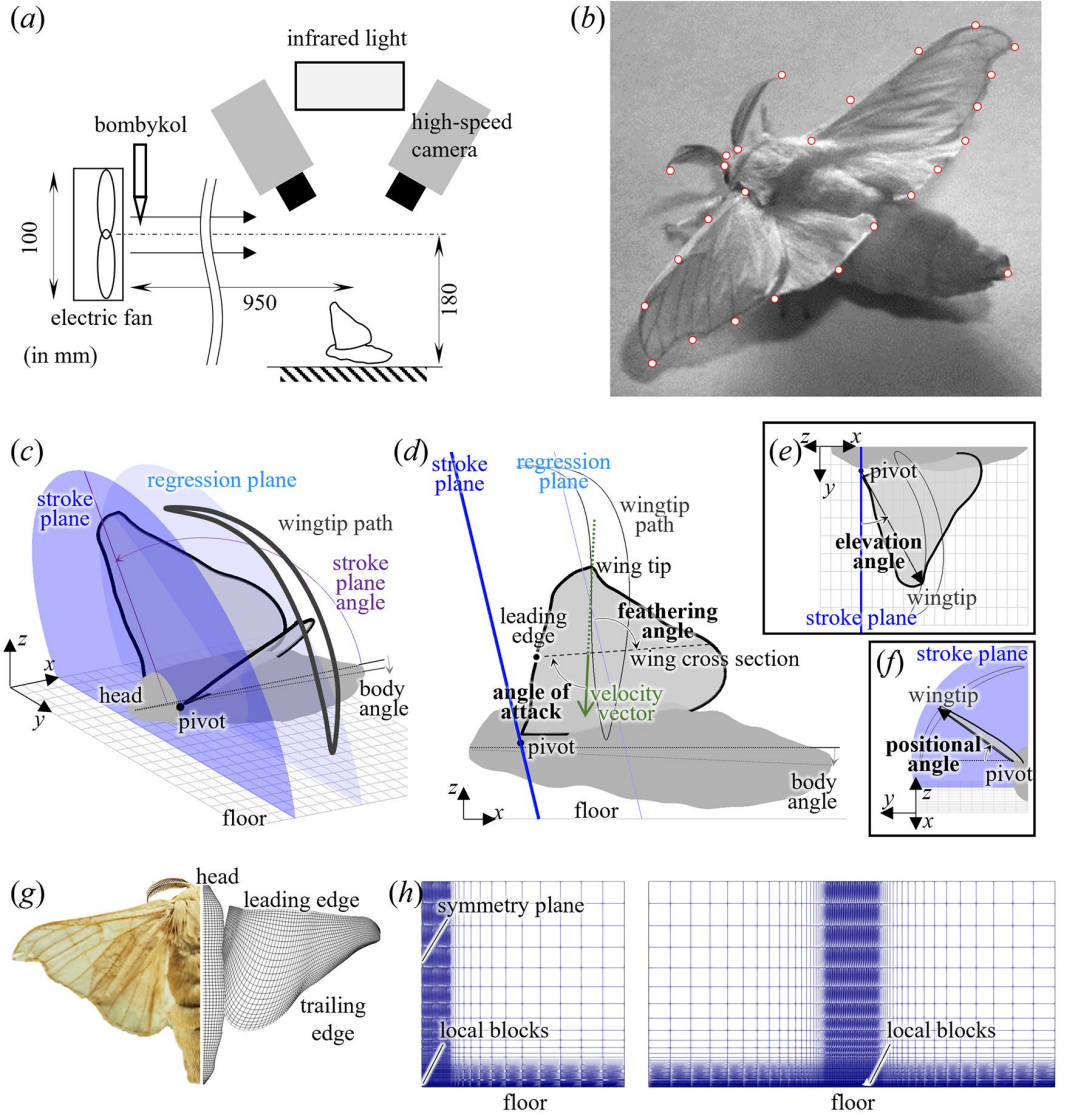
Kinematic reconstruction and morphological model of silkworm moth, *Bombyx mori*. (**a**) Experimental setup for the high-speed filming of fanning silkworm moth. (**b**) An example of the high-speed image of the fanning silkworm moth. Landmarks (yellow circles) in each frame were tracked manually. (**c–f**) Definition of flapping angles. The wing kinematics were defined by positional, elevation, and feathering angles with respect to the stroke plane. (**c**) The stroke plane angle is the angle between the horizontal plane and the plane through which the wing flaps, determined by linear regression of the wingtip trajectory. The stroke plane is the plane through the wing pivot inclined by the stroke plane angle. The body angle is the angle between the horizontal plane and the line connecting the tips of the head and abdomen. (**d**) Side view of the silkworm moth model. The feathering angle is the angle between the wing cross-section and the stroke plane, while the angle of attack is the angle between the wing cross-section and velocity vector. (**e**) The left half of a silkworm moth model viewed from a direction parallel to the stroke plane. The elevation angle is the angle between the stroke plane and the line connecting the wing tip and pivot. (**f**) The left half of a silkworm moth model viewed from a direction normal to the stroke plane. The positional angle is the angle between the horizontal plane and the line connecting the wingtip and pivot in the stroke plane. (**g**) Morphological model of the wing and body of a silkworm moth. (**h**) Frontal (left) and side (right) views of the Cartesian grid to yield the external boundary conditions for the local wing and body blocks sitting at the bottom of the domain.

The fanning of a *B. mori* is elicited by exposing a *B. mori* male to bombykol placed in front of a small electric fan (Fig. 1a; TF85-TFAN, diameter: 100 mm, LADONNA Co., Ltd., Tokyo, Japan) in a room (7.1 m × 5.9 m × 2.6 m) with controlled humidity (60–70%) and temperature (24–26 °C). The wind speed at the recording volume, measured by an anemometer (Anemomaster LITE 6006-DE, Kanomax Japan Inc., Osaka, Japan), is 0.082 m s^−1^, which is insignificant compared to the mean wingtip speed of *B. mori* fanning (2.97 ± 0.72 m s^−1^, *n* = 3). We filmed the fanning of *B. mori* male using two synchronised high-speed cameras (SA3, Photron Ltd., Tokyo, Japan) at 2,000 fps with a shutter speed of 5,000 s^−1^. The cameras were calibrated using the bundle adjustment method^26^. The recording volume was approximately 100 × 100 × 50 mm. Landmarks such as the tip of the head and abdomen, tip and base of the antenna, and wing root and tip (Fig. 1b) were tracked manually using custom-made software written in Python. The three-dimensional coordinates of these landmarks were reconstructed by combining image coordinates. The diagonal distance of the calibration grid on the floor was 52.0 mm, while the distance reconstructed from the image coordinates was 51.9 mm. Thus, the reconstruction error of the recorded volume was approximately 0.2%.

The body, positional (*ϕ*), elevation (*θ*), and stroke plane angles (Fig. 1c–f) were calculated based on the coordinates of the landmarks. The body angle is the angle between a straight line connecting the tip of the head and abdomen and the horizontal plane. The stroke plane angle is the angle from the horizontal plane determined by linear regression of the wing tip path in the *x*–*z* plane (Fig. 1c,d). Stroke plane is the plane through the wing pivot, inclined from the horizontal plane by the stroke plane angle (Fig. 1c,d). The positional and elevation angles were the wing tip angles with respect to the wing pivot within the stroke plane (Fig. 1f) and deviations from the stroke plane (Fig. 1e), respectively.

However, extracting the same point on the wing outline in each camera image was challenging because the wing surface of the *B. mori* was covered with scales and each point was not visible at certain angles. Therefore, similar to previous studies^27,28^, the feathering angle (*α*), which is the rotation of cross-sections around the spanwise axis of the wing (Fig. 1d), was identified by projecting the measured and twisted wing contour in each camera image based on calibration and fitting it to the wing contour defined by clicking on each image. Further details are provided in the Supplementary Material. The angle of attack, which is the angle between the wing and the direction of the incoming flow towards the wing cross-section, has a significant effect on the aerodynamics of the wing. In this study, the angle of attack was determined from the angle between the wing cross-section and wing velocity vector (Fig. 1d) because this study did not consider the environmental wind. The relative wing speed in the direction parallel to the wing surface (*v*_h_) and the component perpendicular to the wing section (*v*_v_) can be determined from the wing angles as follows:

$${{\text{v}}_{\text{h}}} = r(- \dot \phi {{\cos\alpha \cos\theta + \dot \theta \sin\alpha }}),$$

1 $${{\text{v}}_{\text{v}}} = r(-\dot \theta \cos\alpha + \dot \phi {{\cos\theta \sin\alpha )}}$$

where *r* represents the spanwise location of the cross section. Based on the velocity components, the angle of attack (AoA) was calculated as

2 $${\text{AoA}}\text{=}{\text{arccos}}\frac{{\text{v}}_{\text{h}}}{\sqrt{{\text{v}}_{\text{h}}^{2}\text{+}{\text{v}}_{\text{v}}^{2}}}.$$

Because the feathering angle varies along the wing length owing to the twisting of the wing, the angle of attack varies similarly along the wing span.

We reconstructed the kinematic models of three individuals (k1–k3). Each sequence consisted of 3–10 wing beats. First, the wing angles with respect to the body-fixed coordinate system were calculated and averaged over all wing beats. In this study, to simplify the model and save computation time, a symmetry boundary was placed in the sagittal plane and computational analyses were performed only in the left half region of the silkworm moth, considering only the left wing and the left half of the body (see Section "Computational fluid dynamics"). Therefore, the wing angles (Fig. 1c–f) were calculated by averaging the left-and right-wing angles. In some cases, the silkworm moths tilted slightly to either the left or right, causing the wings to slightly interfere with the sagittal plane even when using the averaged wing angles. When the wings interfere with the symmetrical boundary, the model becomes unrealistic, i.e. one wing penetrates the other. Therefore, the wing motions were rotated by approximately 5°–8° around an axis in the horizontal plane through the wing root, directed towards the back of the body. As this rotation is small and limited to only around a horizontal axis, the wing speed is unaffected. Therefore, the effect of this manipulation on the results is considered negligibly small.

### Computational fluid dynamics

In this study, the antennae and environmental airflow were not considered to focus on the effects of wing flapping. Therefore, the antennae were placed virtually in space, and the simulation assumed that the *B. mori* was flapping its wings in still air for simplicity. However, as both antennae and wind may strongly influence on odour sampling, these factors should be considered in future research.

The morphological model of the wing for CFD (Fig. 1d) was constructed using a three-dimensional reconstruction of the wing contour when the wing was nearly flat in an image captured by high-speed cameras. Following the previous studies, the wing thickness was 1% of the wing length with an elliptical cross-section^29^. A morphological model of the body (Fig. 1d) was constructed by photographing the body from the top and sides.

We utilised a bio-inspired dynamic flight simulator based on incompressible, unsteady, and three-dimensional Navier–Stokes equation^29,30^. Reynolds numbers ranged between 1,200 and 2,000, determined using the mean chord length (*c*_m_) of each individual as the representative length and the mean wingtip speed (2*ΦfR*) based on the wingbeat amplitude (*Φ*), wingbeat frequency (*f*) and wing length (*R*) as the representative speed. The simulator employed a multiblock overset-grid method, and the computational domain consisted of a wing, body, and a global Cartesian grid (Fig. 1e). No-slip boundary conditions were adopted on the wing surface, body surface, and floor, whereas the boundary conditions outside the local grids were obtained from the Cartesian grid. Symmetrical boundaries were placed in the sagittal plane, assuming symmetrical motion of the left and right wings. Because the morphology of the wing surface changes over time owing to twisting, grids are generated at every time step. The validation and verification are described in the Supplementary Material.

### Induced flow

The flow field in the background Cartesian grid of the simulation was interpolated to calculate the variation in flow velocity at the antennae and probes of the hot-wire anemometer for validation (see Supplementary Material). The flow field from the simulation was output 50 times per wing beat. At each time step, the flow velocities in three directions at each point were obtained via linear interpolation using the gridded interpolant function in MATLAB (MathWorks Inc.).

### Sampling volume

The sampling volume of the induced flow was quantified using the computed flow field. It is defined as the distribution of the three-dimensional positions of the pheromone molecules reaching the antennae after a particular time (~ 40 wing beats). Therefore, 21 points equally distributed in a straight line from the base to the tip of the antenna were defined as the positions of the antenna, and the streak line was calculated backwards in the direction of time, starting from this series of points at the antenna. We assumed that pheromones are infinitesimally small, weightless particles moving with local flow velocity, so that the streak line represents the trajectory of pheromones arriving at the antenna. The pheromone movement at each time step was calculated as the product of velocity and time step. The flow velocities at all grid points were interpolated in the time direction using third-order Lagrangian interpolation to determine the position of the pheromone at 500 time steps per wingbeat. The velocity at the position of each pheromone molecule at each time step was spatially interpolated using the method described in Section "Induced flow": As the flow distribution varied with the flapping phase of the wings, the molecules was assumed to reach the antennae 21 times at equal intervals during one cycle. The above trajectory calculations were repeated for 40 flapping cycles using repeated data from the 60th flapping cycle of the simulation when the far-field flow had converged sufficiently (see the Supplementary Material).

## Results

### Wing kinematics

**Figure 2:**
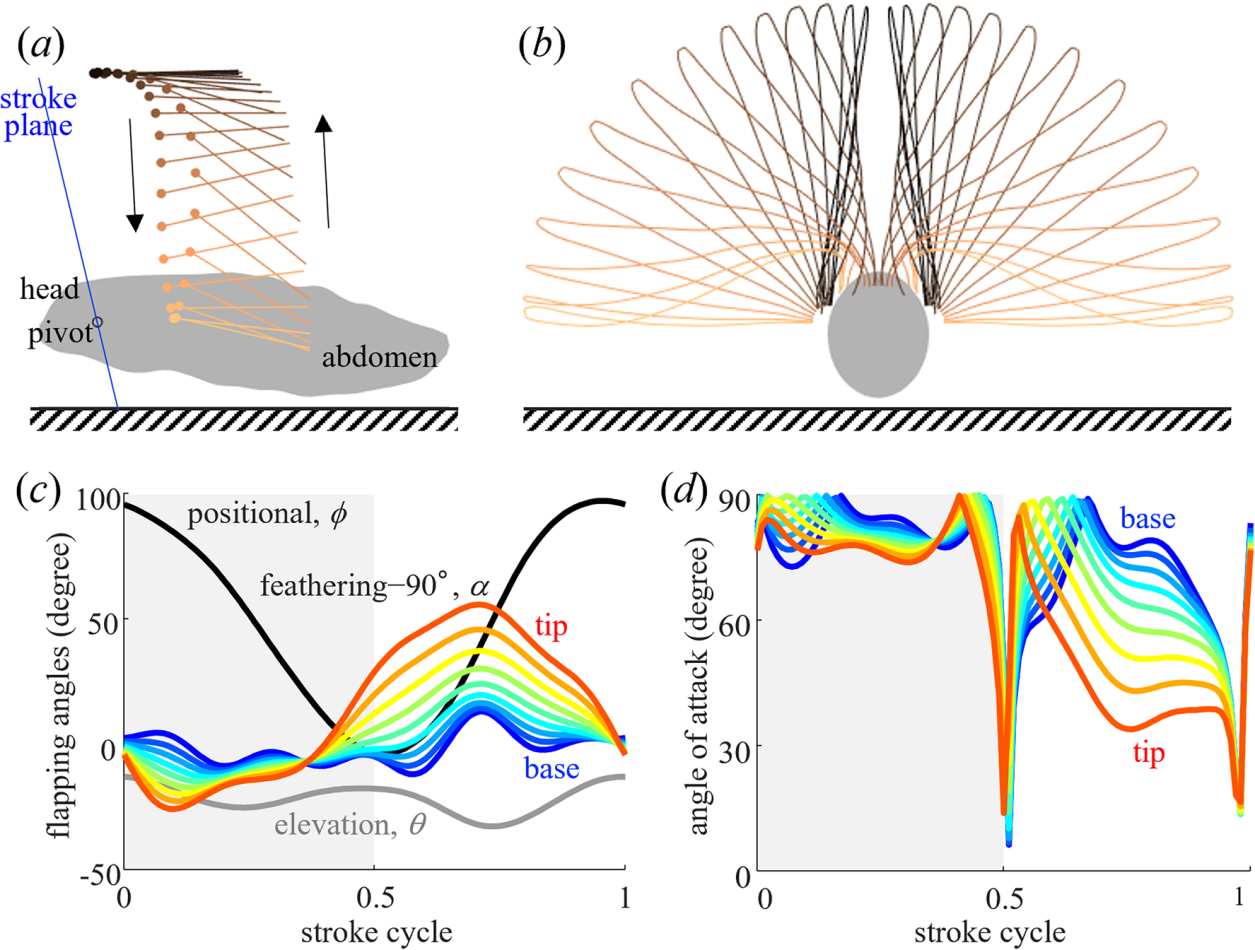
Wing kinematics of silkworm moth fanning. (**a**) Side view of the time series of wing cross sections at 80% of the wing length from the wing root. The blue line represents the stroke plane, and the circles represent the leading edge of the cross sections. (**b**) Frontal view of the time series of the wing outline. (**c**) Time series of the flapping angles with respect to the stroke plane. The stroke cycle on the horizontal axis represents the relative time during a flapping cycle, with 0–0.5 of the stroke cycle (grey-shaded area) corresponding to downstroke and 0.5–1 to upstroke. Note that, to limit the range of vertical axis and to ensure clarity, the feathering angle is subtracted by 90°. (**d**) Time series of the angle of attack. The grey-shaded area represents the downstroke. See Fig. 1 for the definition of flapping angles.

The flapping kinematics of the fanning *B. mori* were measured and described in the same manner as for flying insects^31^. The measured parameters are listed in Table 1. The body angle was close to horizontal, and the stroke plane was approximately vertical (Fig. 2a). The elevation angle was always negative, i.e. the wing flap posteriorly relative to the wing root (Fig. 2a). During pronation, a motion similar to clap-and-fling^32^, where the wings approach and move away from each other on the dorsal side of the body, was observed (Fig. 2b). In the late downstroke, the wingtips were closer to the ground and could not flap to the ventral side of the body, compared to that of other lepidopterans^33–35^. In some cases, the left or right wing touch the ground during supination. In this study, the flapping angles of the left and right wings were averaged; therefore, in the data shown in Fig. 2, both wings were distant from the ground during supination. The feathering angles varied less near the wing root (Fig. 2c). However, near the wing tips, where the relative velocity was higher and had a significant influence on the generation of aerodynamic forces and flow fields, the variation in the feathering angle was larger, particularly during the upstroke, owing to the passive twist of the wing. The angle of attack remained approximately 90° during the downstroke and decreased during the upstroke (Fig. 2d).

**Table 1:** Summary (mean ± std, *n* = 3) of the morphological and kinematic parameters of the individuals for kinematic reconstruction.

Wing length (mm)	17.3 ± 0.6
Mass (g)	0.237 ± 0.038
Stroke plane angle (degree)	99.5 ± 15.6
Body angle (degree)	− 0.6 ± 3.1
Wingbeat amplitude (degree)	98.0 ± 9.4
Wingbeat frequency (degree)	48.9 ± 8.6
Mean elevation angle (degree)	− 23.8 ± 2.4

### Aerodynamics, energetics, and near-field aerodynamics

**Figure 3:**
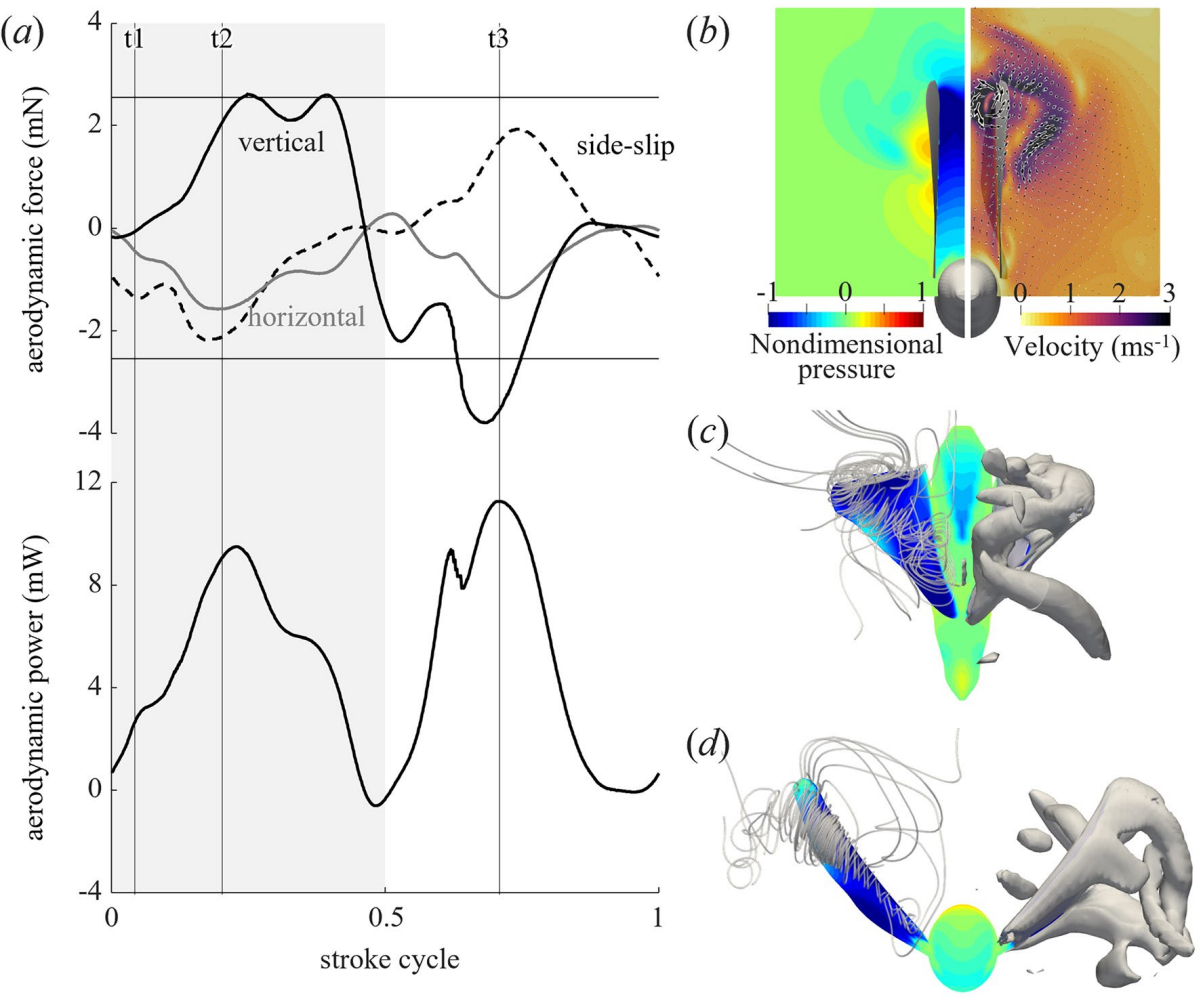
(**a**) Time series of aerodynamic forces and power. The weights of the silkworm moth (k1, 0.26 g) are shown by horizontal lines for comparison. Grey-shaded area represents the downstroke. The side-slip force is cancelled when the left and right wings are added; thus, the values for one wing are shown in the figure. (**b–d**) Simulated instantaneous flow field around fanning silkworm moth viewed from (**b**, **d**) front or (**c**) top at t1–t3. (**b**) Pressure (left) and velocity (right) on the frontal plane on the wing pivot. (**c**,**d**) Instantaneous streamlines (left) and iso-Q surfaces (right) with surface pressure. The time steps (t1–t3) are represented by the vertical line in (**a**).

Despite their flightlessness, *B. mori* generate large aerodynamic forces by fanning their wings (Fig. 3a; Supplementary Fig. 5). Owing to the aforementioned kinematics, the aerodynamic forces are large in the direction vertical to the floor, and in some individuals, the peaks reach their own weights. In contrast, the maximum horizontal aerodynamic force is approximately 50% of the body weight, with a cycle average of 5.0–12.8% (0.21 ± 0.11 mN, *n* = 3) of the body weight. Flying insects use the aerodynamic forces normal to the stroke plane to support their own weight or move forward by cancelling the drag on the body^36^. For the *B. mori*, the stroke plane is close to the vertical direction; therefore, the horizontal forces correspond to the vertical forces of flying insects. During the downstroke, the feathering angle of the *B. mori* varied less along the wingspan, and its angle of attack was larger than that during the upstroke (Fig. 2c, d), which increased the difference between the patterns of vertical and horizontal forces during the downstroke and upstroke. The fanning requires a maximum power (Fig. 3a) of 4.8 mW with a cycle average of 2.7 ± 1.9 mW (*n* = 3).

The aerodynamic forces described above are generated mainly by aerodynamic mechanisms similar to those of other flying insects^37^. During the downstroke and upstroke, when the aerodynamic forces generated were the greatest, a leading-edge vortex and an associated strong negative pressure were generated (t2, t3; Fig. 3c,d). When the wings separated during pronation, strong airflow and negative pressure were observed between them (t1, Fig. 3b). Therefore, the side-slip forces and small peaks in the horizontal forces during pronation (t1) were caused by clap-and-fling. Vortices and strong negative pressure were also observed near the trailing edge of the wing when large aerodynamic forces from the downstroke occurred (Fig. 3c), suggesting that the clap-and-fling contributed to the generation of large forces during pronation and over the downstroke duration.

### Induced flow and sampling volume

**Figure 4:**
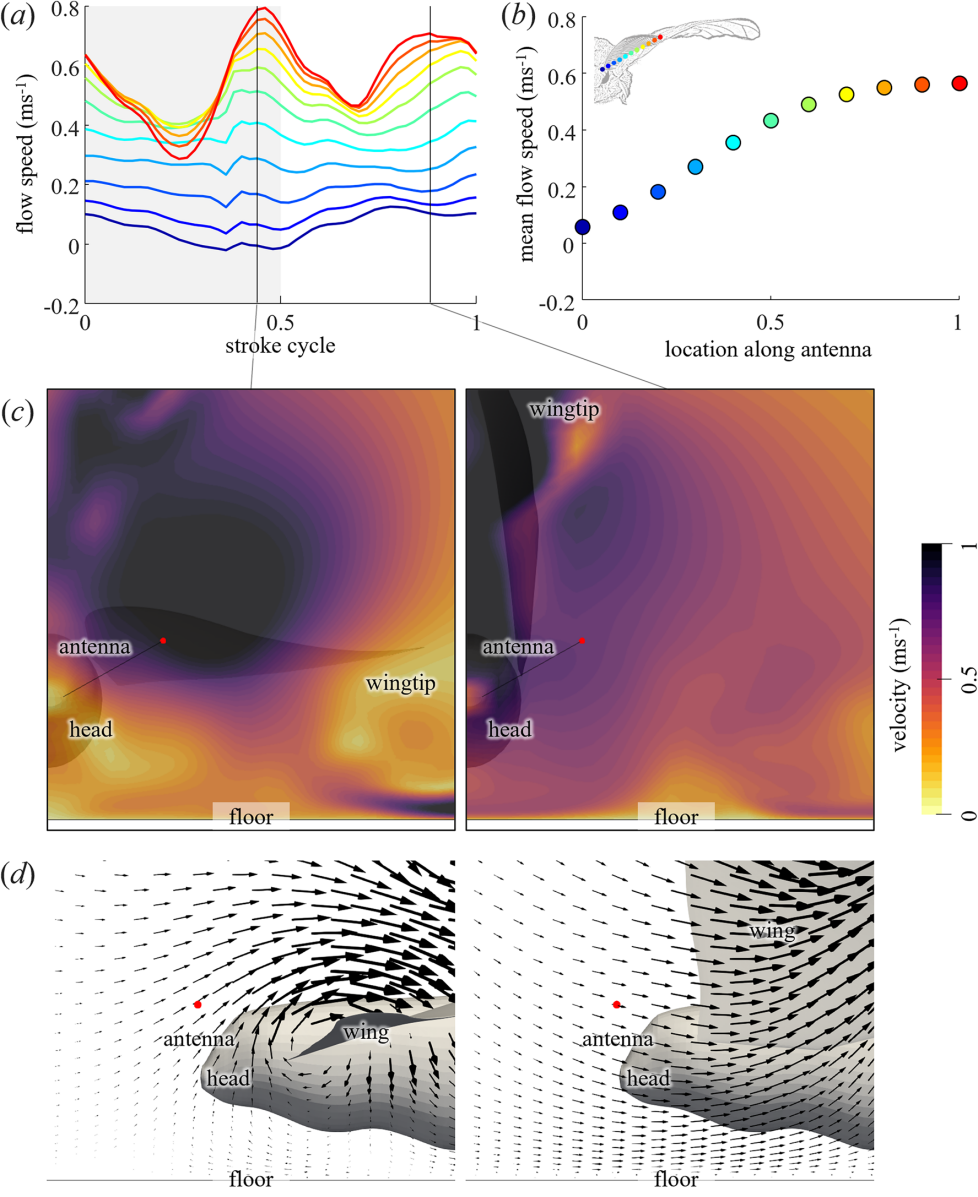
Induced flow around the antenna by the fanning of a silkworm moth. (**a**) Time series and (**b**) cycle average of the induced flow speed at the antenna. The colours represent the location along the antenna, as shown in the insets in (**b**). (**c**) Snapshots of the velocity on the frontal plane at the tip of the antenna (red dot) when the flow velocity is maximised. (**d**) Side view of the vector field on the vertical plane at the tip of the antenna (red circle).

The flapping wings induce an airflow towards the antennae. The flow velocity at the antennae oscillated synchronously with wing flapping (Fig. 4; Supplementary Fig. 6). In some cases (k1 and k3; see Supplementary Fig. 6), the peaks were observed for both pronation and supination; however, in another case (k2; see Supplementary Fig. 6), the peaks were observed only for pronation. In other words, the airflow near the antennae oscillates at either the wingbeat frequency or twice the wingbeat frequency. The variation is attributable to variations in wing kinematics and antenna positions. As the wing rotated around the root and its speed increased towards the wingtip, the mean and amplitude of the airflow increased from the root to the tip of the antennae (Fig. 4b,c). The airflow at the antenna changes direction between the downstroke and upstroke (Fig. 4d). When the flow speed is maximised, the flow produced by the downstroke is directed upward (left in Fig. 4d), while the flow produced by the upstroke is directed downward (right in Fig. 4d). The airflow oscillated at the tip of the antennae at an average of 0.35 ± 0.18 m s^−1^ (*n* = 3) with an amplitude of 0.34 ± 0.22 m s^−1^ (*n* = 3) and at the middle of the antennae at an average of 0.24 ± 0.19 m s^−1^ (*n* = 3) with an amplitude of 0.20 ± 0.10 m s^−1^ (*n* = 3). The flow velocities at the upstream of the *B. mori* antennae were reported to be 0.3–0.4 m s^−1^^16^. Although the detailed location of the measurement points relative to the antennae is unknown, the simulated results of this study, particularly near the virtual antennae tip, agree well with the measurements. Owing to the distance between the wing and antennae, the timing of the maximum airflow was delayed in phase by approximately a quarter of a cycle compared to the timing of the maximum wing speed at the downstroke and upstroke.

**Figure 5:**
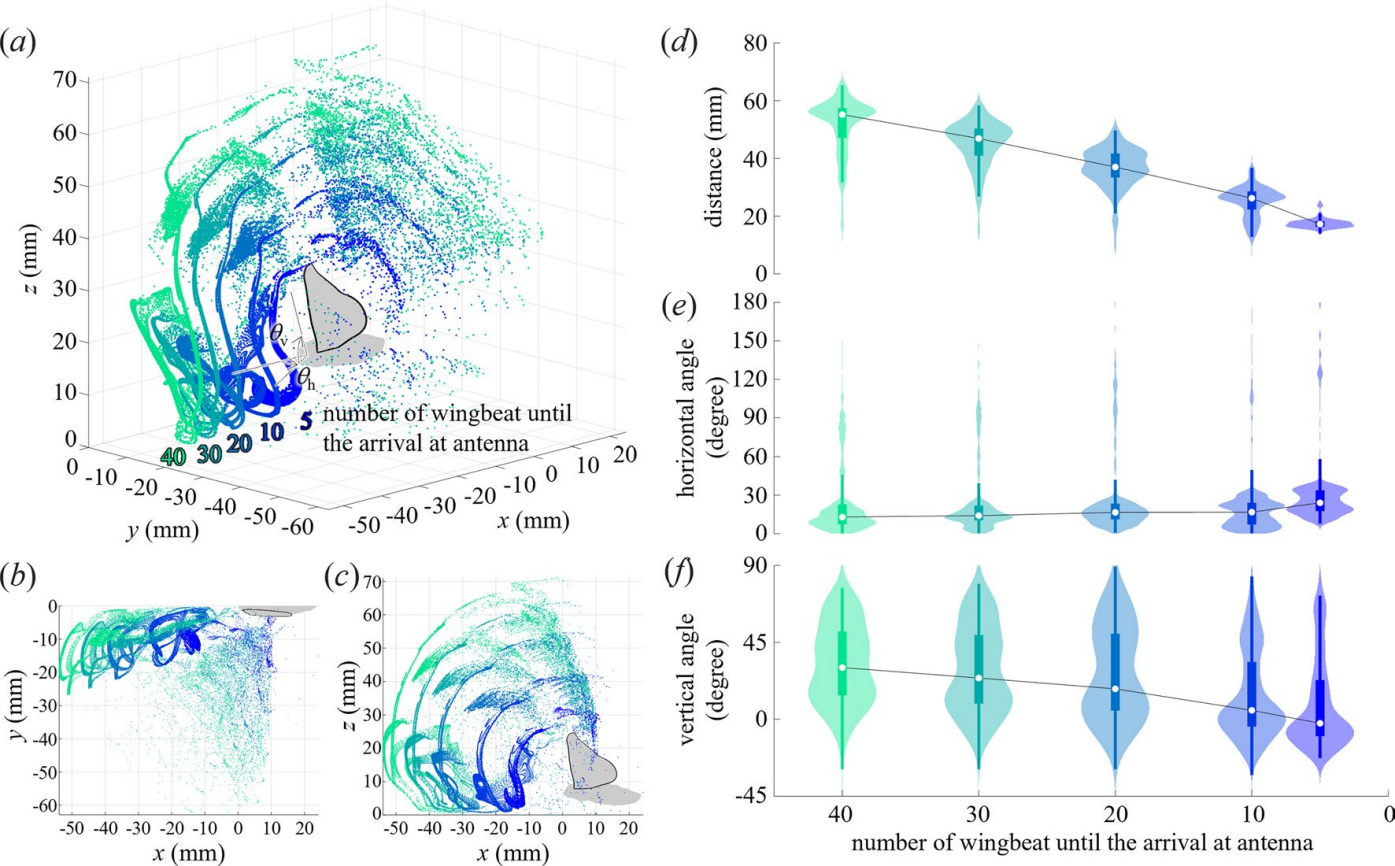
Distribution of the particles entrained by fanning. (**a**) Three-dimensional, (**b**) top and (**c**) side views of the distribution of particles that reach the antenna after 5–40 wingbeats. Time series of the violin plot of the (**d**) distance, (**e**) horizontal and (**f**) vertical angles of the particles with respect to the head of the silkworm moth. The particle’s arrival time at the antenna is set to zero. White dots, thicker and thinner lines represent the median, the 25th to 75th percentile ranges, and those of minimum and maximum without outliers, respectively.

*B. mori* guide the airflow, and hence particles in the air, towards their antennae from a limited range from in front of the moth (Fig. 5; Supplementary Fig. 7). We derived the sampling volume by considering the line connecting the antenna base and tip as the virtual antenna and simulating the particle motion in the reverse direction in time by releasing particles from 21 equally spaced points along the line, 21 times constantly over a wingbeat. The simulated motion of particles indicates that pheromones reach the antennae from approximately 20–30 mm for 10 wingbeats and 40–50 mm for 20 wingbeats (Fig. 5d). The *B. mori* guides the particles from a wide range surrounding the body by fanning (Fig. 5a–c); however, most of the particles arrive in the vicinity of the antenna from a limited range within approximately 30° (0–30°) horizontally with a single wing (Fig. 5e) or 60° with the left and right wings. The distribution of horizontal angles did not vary with time until arrival at the antenna. A similar trend was observed in other individuals (Supplementary Fig. 7); however, the captured particles were distributed in a narrower range than in the individuals shown in Fig. 5. In the vertical direction (Fig. 5f), the particles reaching the antennae of all individuals exhibited a relatively wide, almost bimodal distribution because the direction of the airflow changed vertically during the downstroke and upstroke, resulting in an airflow drawn from below or above the antennae (Fig. 4d). Therefore, the distribution of pheromones reaching the antennae does not change significantly over time, except for distance, which is in a limited area in front of the moth.

## Discussion

### Wing kinematics and aerodynamics of flightless silkworm moths

High-speed photogrammetry of *B. mori* fanning revealed differences in wing kinematics compared with other flying insects. In flying insects, the wingtips oscillate in the stroke plane at approximately the same height as the wing root, and the elevation angle varies at around 0°^33,34^. In the *B. mori*, the elevation angle was always negative, and the wings flapped posteriorly (Fig. 2a–c). Negative elevation is inefficient during flight because the lift provided by the wings is directed towards the outside of the body. Owing to domestication, the ratio of the flight muscle mass of the *B. mori* to its own mass is smaller than that of wild species, with irregular flight muscle morphology and the absence of a perimysium in the flight muscles^8^. These muscular changes presumably caused them to flap their wings as described above, in contrast to the way performed by flying insects. The wings of domesticated *B. mori* are also more flexible than those of wild silkworm moths^8^.

In this study, passive twisting of the *B. mori* wing was high during the upstroke (Fig. 2c,d). The asymmetry of the twist at the downstroke and upstroke was owing to the asymmetry in the flexibility of the *B. mori* wings. The *B. mori* wing is similar to that of flying insects in terms of wing stiffness anisotropy; anisotropy between the ventral and dorsal stiffnesses of the wing has been observed in the hawkmoth *Manduca sexta*^38^ and butterflies^39^.

Despite the differences in wing kinematics, *B. mori* produce a leading-edge vortex similar to those of flying insects^37,40^ by flapping their wings. Using this leading-edge vortex, a *B. mori* can generate large aerodynamic forces on a scale comparable to its weight (Fig. 3a). Furthermore, the *B. mori* can move using the aerodynamic forces generated from its flapping wings, even when its legs are removed^41^. Fanning requires power to overcome aerodynamic forces; however, the power (cycle average of 4.8 mW for an individual weighing 0.26 g) is relatively lower compared to that of a butterfly of similar mass, for example, the flying painted lady butterfly *Vanessa cardui* (0.29 g, 10.5 mW)^34^. However, owing to the large angle of attack (Fig. 2d), the variation in the vertical force was large, and the averaged thrust was low (Fig. 3a). Although fanning would assist propulsion, the propulsion by fanning may not be energy-efficient, as it consumes unnecessary energy to generate large aerodynamic forces in directions unrelated to locomotion.

### Three-dimensional induced flow

Airflows induced by fanning varied both temporally and spatially. The flow velocity owing to fanning strongly influences the detection of pheromones by the antennae. If the velocity is low, the boundary layer at the sensilla is comparable to the distance between the sensilla; therefore, the airflow is diverted around the antennae, and the air carrying the pheromone does not reach the sensilla. In the pectinate antennae of the silkworm moth, a high level of leakiness must be maintained to ensure that the flow can pass through the gaps in the sensilla^16,42^. However, if the flow is too fast, the capture efficiency is reduced because the pheromones do not have time to diffuse to the surface of the sensilla. Previous studies indicated that an optimum flow velocity exists at the antennae^43,44^. In this study, we show through the simulation-based approach that the flow velocity owing to the flapping of the wings fluctuates over time, as shown by measurements using a hot-wire anemometer in a previous study^16^, in a manner similar to that for flying insects^17,18^. The flow velocity owing to fanning is higher at the wingtip than at the wing root; thus, the flow velocity is distributed on the antennae. However, it is unclear how this spatial distribution of flow velocities affects sampling at the antennae and must be considered when evaluating the aerodynamics of the antennae because the distribution can affect local leakiness. In flying insects, the inflow velocity into the antennae is determined by the sum of the flight speed and the velocity induced by wing flapping. Therefore, regarding insects, the wind speed in the habitat and their flight speed must be considered, including the induced flow from the flapping wings when guiding the airflow to the antennae.

### Directional sampling volume

*B. mori* can direct distant and frontal pheromones to their antennae by flapping their wings (Fig. 5d–f). The anisotropy of the sampling volume is as crucial as the precise detection of odour molecules. The *B. mori* rotates and localises the direction from which the odour arrives^10^; similarly, a robot without a wind sensor can determine the direction with an algorithm with rotation^45^. The anisotropy in the sampling volume enables fanning *B. mori* to determine the direction faced by the bodies of the odour plume when they detect the odour. The difficulty of *B. mori* in finding females when their wings are excised^46^ is probably because of their inability to determine the direction of the odour source without fanning in low-wind environments.

The sampling volume size may also influence the strategy of *B. mori* males in their search for females. A previous study^10^ reported that *B. mori*, when exposed to pheromones for a short period (0.1 s) once every 4 s in 0.5 m s^−1^ wind, rotated about 68° ± 5° over 1 ± 0.1 s in the opposite direction to that in which they were rotating. As shown in Fig. 5a, pheromones in space reach the antennae after some time, depending on the relative position of the pheromone and the antennae. Therefore, if a pheromone is detected while rotating and is not detected immediately after that, it is likely that the odour plume went outside the sampling volume owing to the rotation of the body, and the *B. mori* male needs to scan the space again by rotating in the opposite direction. If the odour plume is already outside the sampling volume, it would be possible to scan the space efficiently by turning back as much as the size of the sampling volume. The rotation angle of the *B. mori* obtained in the previous study is close to the sampling volume size obtained in this study (60°), and the sampling volume is possibly considered in the odour source localisation of *B. mori*.

A combination of advection and diffusion determines the trajectory of molecules in a fluid. For simplicity, only the advection effects were considered in this study. A previous study examining the effect of various factors, including the Schmidt number, on the concentration distribution of odour molecules near flapping flying insects reported that the molecular diffusion effect is greater when the Schmidt number is around 0.5, while the advection effect is greater when the Schmidt number is around 10^47^. The advection effect is considered dominant at the Schmidt number of bombykol in air (6.0; see 2.1), but the diffusion effect may not be negligible. Therefore, the particle volume distribution (Fig. 5) could be widened by diffusion, and simulations considering the effects of particle diffusion will be necessary to accurately quantify the sampling volume in the future.

The size and anisotropy of the sampling volume are also crucial when implementing it odour sensors in robots for odour-source localisation. Multicopter-type drones are often used in flying robots, and the flow field can be symmetrical in drones with symmetrically arranged rotary blades. Therefore, without a casing, as in previous studies^45,48^, the anisotropy of the sampling volume is insignificant, and wind sensors must be installed to obtain directional information^49^. Despite some terrestrial robots improving anisotropy by utilising fans^50^, mounting devices is not desirable owing to the limited payload of flying robots. Therefore, odour-source localisation using robots requires further consideration of configurations to draw in odours from greater distances in an anisotropic manner. Adopting alternative forms of propulsion, such as flapping wings^51^, is one solution; however, the performance of odour source localisation with multicopter drones can also be improved by comprehensively considering the arrangement of odour sensors on the drone and the location and angle of the rotor blades.

### Limitation of the study

In this study, the antennae and environmental airflow were not considered to focus on the macroscopic flow field induced by fanning. However, particles reaching the virtual antennae may not reach the sensors on the antennae because of the complex flow pattern in the vicinity of the antennae^16^. The antennae of *B. mori* are relatively large and can affect the airflow distribution produced by wing flapping. Their antennae are multiscale microstructures, and considering their interaction with the surrounding airflow is challenging, although important towards obtaining a comprehensive understanding of insect behaviour. Because odours are carried by wind, airflow in the environment, including turbulence^47^, can also affect odour source localisation by strongly distorting the sampling volume if the flow velocity is close to or exceeds the simulated flow velocity. To cope with this complexity, organisms localise odour sources by integrating wind direction estimated from mechanosensation and visual anemotaxis^52,53^. Wind sensors have also been proposed as effective for odour-source localisation in robots^49^. Future studies should also consider the effect of environmental airflow and turbulence on the sampling volumes of insects and robots to better understand the odour-source localisation strategy.

## Conclusions

The silkworm moth, *Bombyx mori*, has been used as a model insect to study odour source localisation. Studies on *B. mori* have provided crucial insights into the morphology of insect antennae and odour source localisation algorithms. Owing to domestication *B. mori* do not fly; hence, they modulate the flow field near the antenna by fanning, which influences odour source localisation. However, the mechanism by which *B. mori* induce airflow and the effect of these airflows on the entrainment of pheromones in antennae have not been thoroughly investigated. In this study, the aerodynamics of *B. mori* fanning were investigated by measuring the wing kinematics of a *B. mori* and conducting numerical simulations. These results provide new insights into odour source localisation in *B. mori* and offer new design guidelines for odour source search robots. Future research in these directions can further refine the understanding of insect olfaction and inspire innovations in robotics and sensor technologies.

### Supplementary Information

Supplementary Information.

## Data Availability

The datasets used and/or analysed during the current study available from the corresponding author on reasonable request.
