# Supplementary material for: Olfactory sampling volume for pheromone capture by wing fanning of silkworm moth: a simulation-based study
Source: Sci Rep. 2024 Aug 2;14:17879. doi: 10.1038/s41598-024-67966-y (PMC11297250; doi:10.1038/s41598-024-67966-y)
Supplement: Supplementary file 1 — Supplementary Information. [file 41598_2024_67966_MOESM1_ESM.pdf]

Supplementary material for "Olfactory sampling volume for pheromone capture by wing fanning of silkworm moth: a simulation-based study"

S1. Kinematic reconstruction

The three-dimensional reconstruction of landmarks is described in Section 2.2. The reconstruction of wing outlines and feathering angles is described in detail in this section. In each frame, three points on the leading edge and five points on the trailing edge were manually digitised to extract the wing outline from each image. The distribution of the feathering angle along the spanwise axis of the wing was adjusted by fitting the wing root and tip of the wing morphology model to the measured three-dimensional coordinates. The spanwise distribution of the feathering angle α is assumed as follows:

$$\alpha(r) = \alpha_{\mathrm{b}} + \alpha_{\mathrm{t}}\left(\frac{r}{R}\right)^{n}, \tag{S1}$$

where α_b and α_t are the feathering angles at the wing root and wing twist, respectively; r is the distance from the wing root; and R is the wing length. Using α_b, α_t, and n as variables, the distances between the model outline and the leading and trailing edges in each image were minimised using the optimisation toolbox in MATLAB (fmincon). In all frames, the average minimum distance between the model outline and the outlines in the image was approximately 3.2 pixels, indicating a sufficiently good agreement. Examples of fitting are shown in Supplementary Figure 1. Equation (S1) is suitable for the above minimisation because it enables the morphology to be expressed with fewer variables; however, n does not vary smoothly over time. Despite a good agreement being obtained near the wing tips, the relative movement of the fore and hind wings was not considered, resulting in large deviations in the outlines near the wing roots (see Supplementary Figure 1*c*, *d*). To ensure smooth variation in the parameters and enable interpolation in time, the distribution of the feathering angles was fitted again with a polynomial at each time step as follows:

$$\alpha(r) = \alpha_{\mathrm{b}} + \alpha_{\mathrm{sq}}\left(\frac{r}{R}\right)^{0.5} + \alpha_{1}\left(\frac{r}{R}\right) + \alpha_{2}\left(\frac{r}{R}\right)^{2}. \tag{S2}$$

A feathering angle ranging from 40 % to 90 % of the wing length was used for the distribution of feathering angles defined by Equation (S1). For subsequent numerical analyses, the positional angle, elevation angle, α_b, α_{sq}, α_1, α_2 of the feathering angles were interpolated using a fifth-order Fourier series. The measured flapping angles and angles of attack for all individuals (k1–k3) are shown in Supplementary figure 2.

S2. Validation and verification

The simulation results were compared with the measured airflow velocities to validate the reliability of the simulations. A hot-wire anemometer (Smart CTA 7250, Kanomax Japan Inc.) was placed near the antennae of the tethered *B. mori* to measure the airflow induced by the flapping wings. The hot-wire anemometer was calibrated to measure air velocity (0–1 m s^{-1}) with high accuracy using the voltage at the probe and measurements from a calibrated anemometer (Climomaster, Kanomax Japan Inc.) obtained in a small suction-type wind tunnel with a PC fan (Supplementary Figure 3*a*, *b*). A hot-wire anemometer and two high-speed cameras, calibrated as described in Section 2.2, were synchronised to simultaneously capture images of the flapping wings. A total of 10–57 wingbeats were recorded. The three-dimensional coordinates of the head and tip of the abdomen, wing tips, wing roots, and the probe of the hot-wire anemometer were extracted from the high-speed images to measure the position of the hot-wire anemometer relative to the body (Supplementary Figure 3*c*). The flow velocity in each flapping phase was calculated based on the time series of wing angles calculated from the coordinates of the wing tips. The measured speeds were filtered using a Butterworth filter with a cutoff frequency of 500 Hz. The wind speed of the fan, which was estimated using a hot-wire anemometer, was approximately 0.082 m s^{-1}. Subsequently, the estimated speed was subtracted from the measured speed. As summarised in Supplementary Table 1, individuals used for flow measurement (wing length: 23.9±0.1 mm, n = 4) were larger than those for kinematic reconstruction (17.3±0.6 mm, n = 3).

Therefore, the position of the hot-wire anemometer and flow velocity were normalised by the wing length and mean wingtip velocity ($2\Phi Rf$, where Φ, R, and f are the wingbeat amplitude, wing length, and wingbeat frequency, respectively). As shown in Supplementary Figure 3*d*, the measured flow velocities and simulation results are in very good agreement in terms of phase and amplitude; therefore, the simulation results are reliable.

The self-consistency of the grid was tested by comparing the results from the finer mesh and time steps (finer) used in this study with those from coarser meshes and time steps (coarse) and finer meshes and time steps (finest) (Supplementary Table 2). The total aerodynamic forces and powers and the flow velocity (60 th wingbeat cycle) from each mesh are shown in Supplementary Figure 4. The flow velocity was sampled in a vertical plane 40 mm from the head of the *B. mori*. Despite the large differences between the coarse mesh and other two cases, the finer and finest cases showed reasonable agreement. Therefore, fine grids and timesteps were used in this study. The time-series of the flow velocity at a point 40 mm from the head of the silkworm moth is shown in Supplementary Figure 4*c* as an example of the time history of the far-field flow. As the flow velocity is well converged after approximately 60 cycles, the repetition of the flow field at the 60th cycle was used to calculate the particle trajectories.

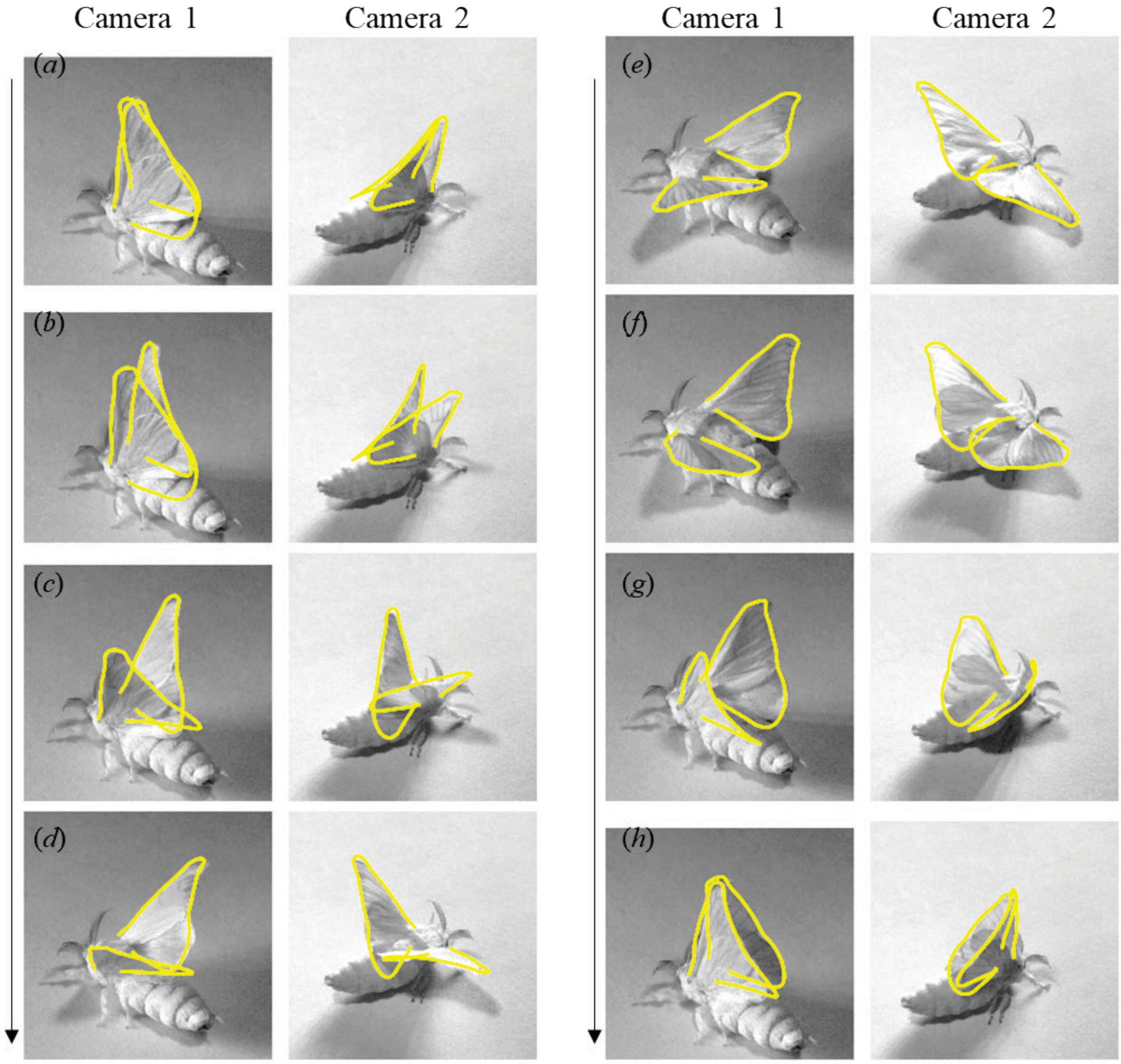

Supplementary Figure 1 High-speed images cropped to 400×400 pixels and outlines of the fitted wing model (yellow lines), showing one wingbeat of images every four frames.

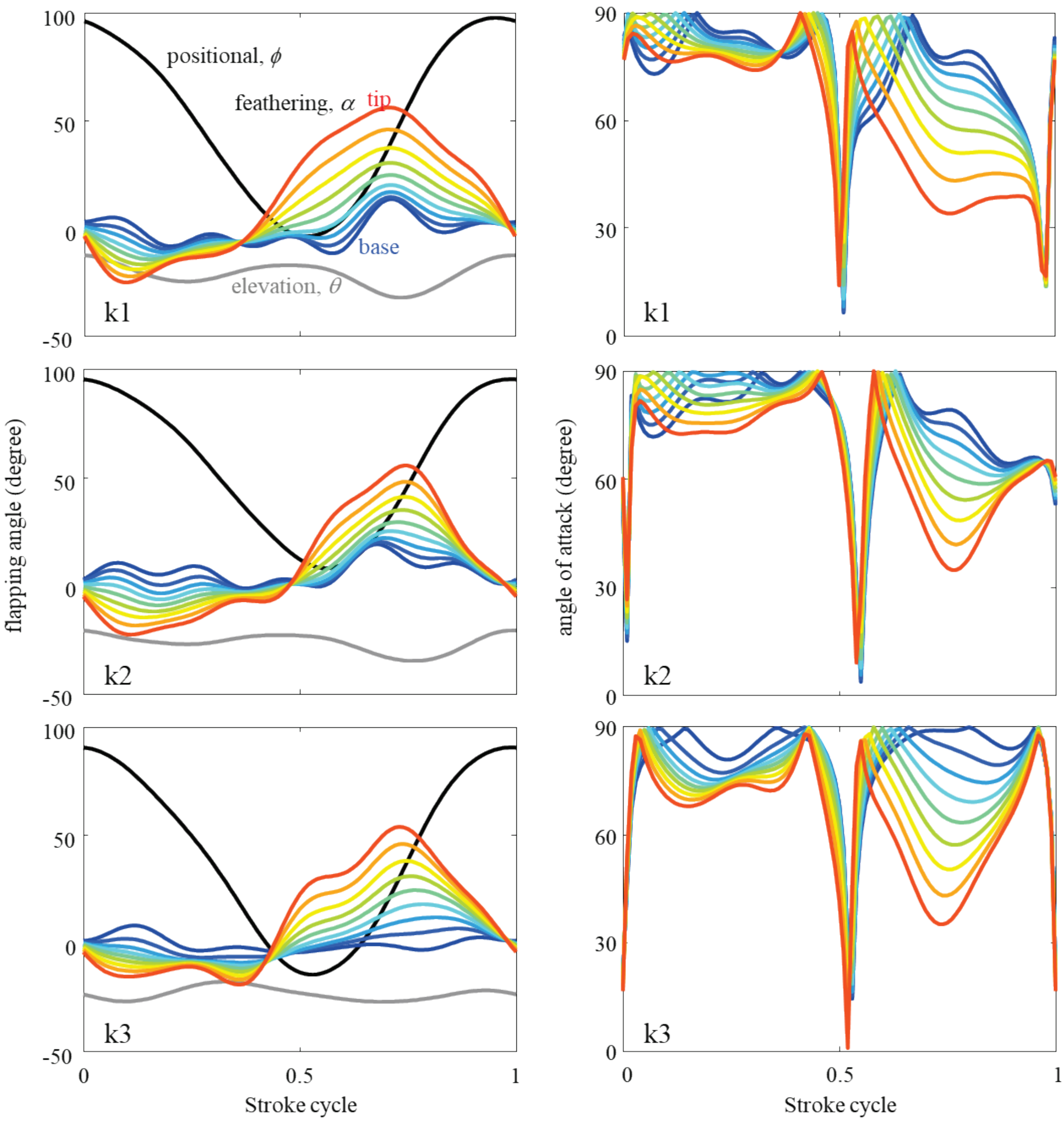

Supplementary Figure 2 Time series of the (left) positional (black), elevation (grey) and feathering (coloured) angles and angle of attack of three individuals ((top) k1, (middle) k2 and (bottom) k3). The feathering angles and angle of attack were coloured by the spanwise location from base (blue) to tip (red). See Figure 1 for the definition of flapping angles.

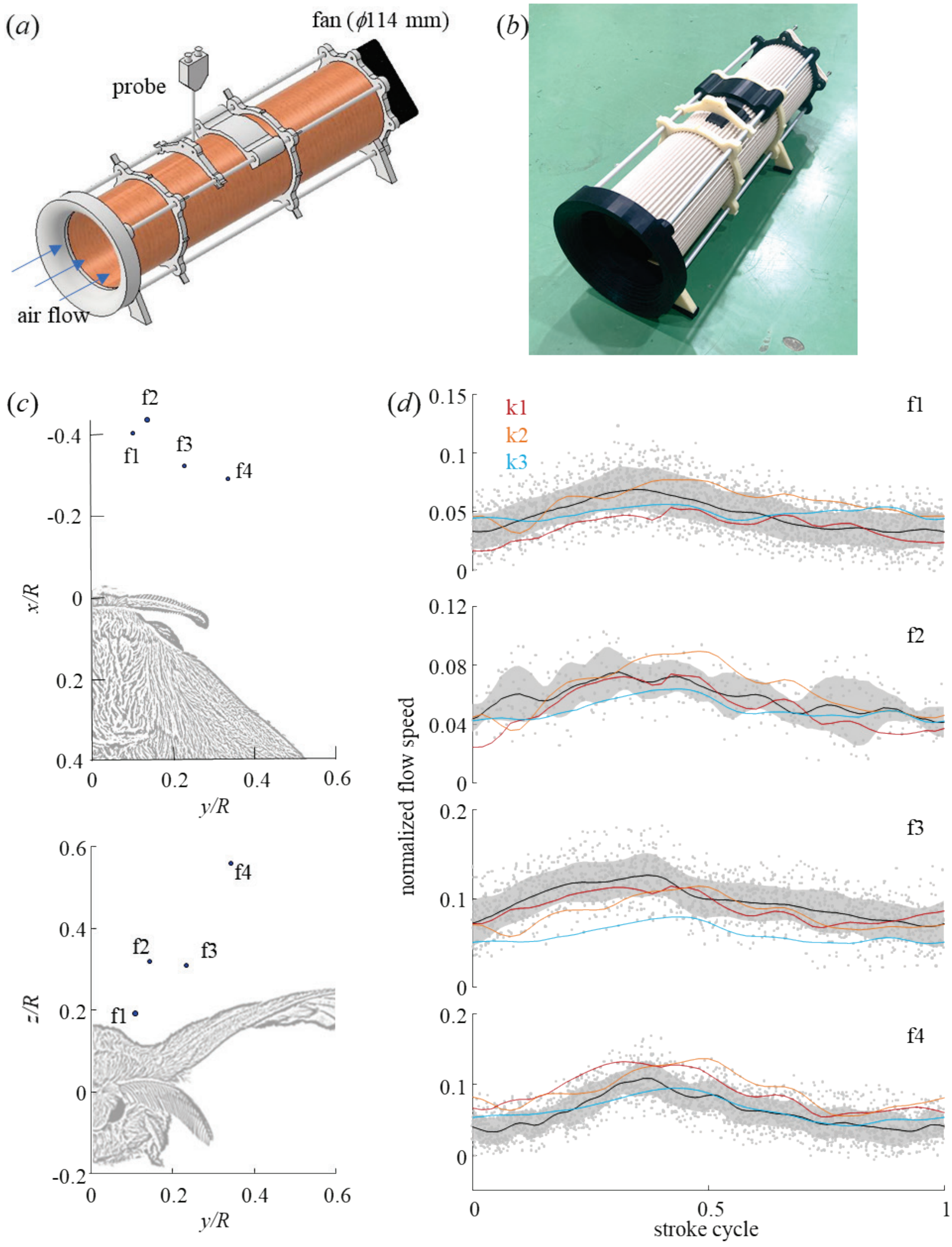

Supplementary Figure 3 Flow measurements for the validation of CFD results. (*a*, *b*) Low-speed suction-type wind tunnel for the calibration of the hot-wire anemometer. (*c*) The location of the probes for flow measurement is normalised by the wing length of each individual (f1–f4). The origin is defined as the tip of the head. (*d*) Time series of the flow velocity at the probes normalised by the mean wing tip velocity. Filtered speed, mean speed, and standard deviations derived from the measurements are represented by grey dots, black lines, and a grey area, respectively. The coloured lines represent the simulated flow speeds of different individuals.

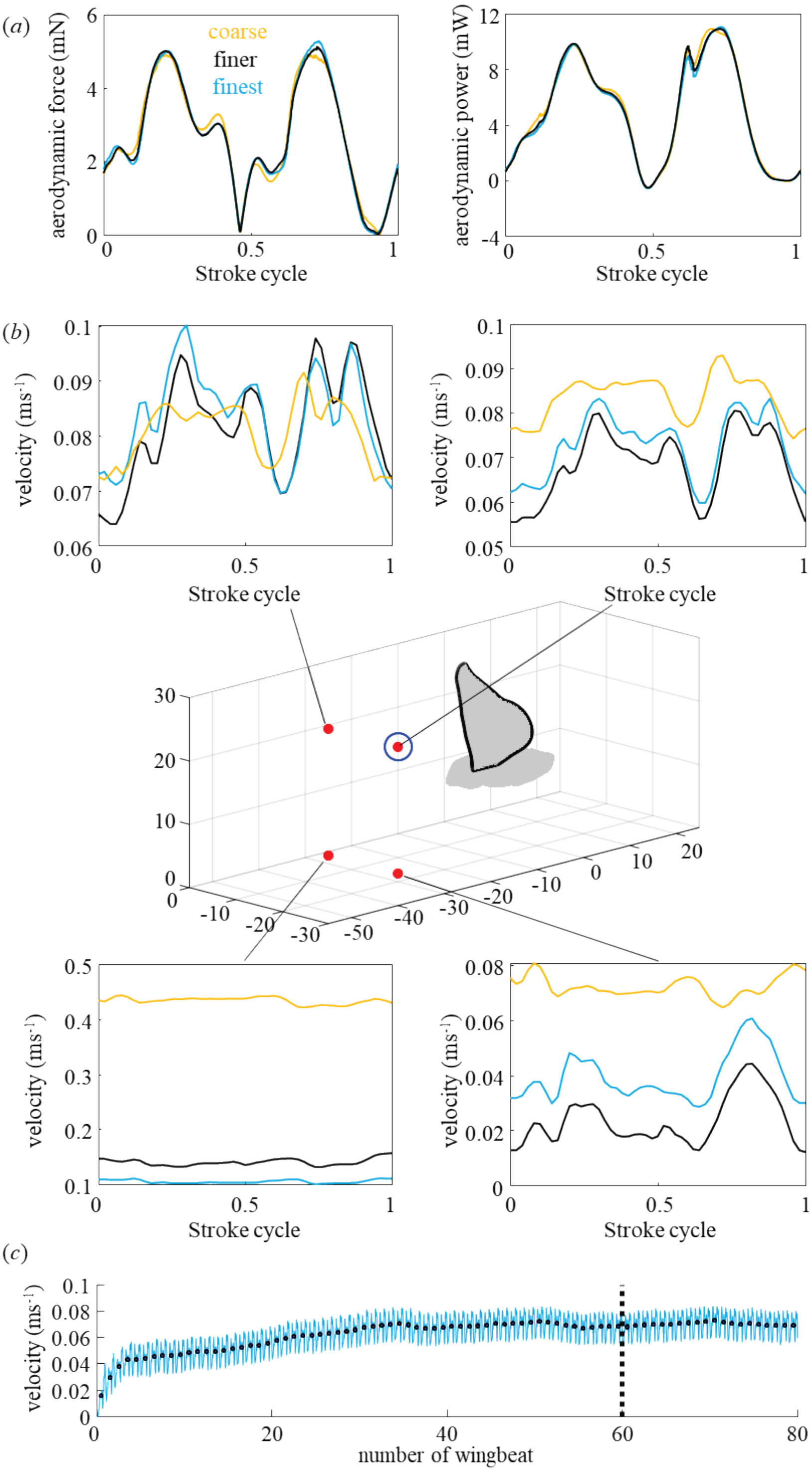

Supplementary Figure 4 Verification of the computational grid and flow field convergence. (*a*) Time series of the resultant aerodynamic force and power; and (*b*) flow velocity simulated by coarse, finer, and finest meshes and time steps. The flow velocity in (*b*) was sampled at the vertical plane placed 40 mm from the head of the silkworm moth model. In the central diagram in (*b*), the a silkworm moth model and the position of the flow velocity are indicated by red dots, and the corresponding figure of time series of the flow velocity is connected to the red dots by black lines. (*c*) Time-series of flow velocity at the furthest point from the model (the blue circle in (*b*)) among the sampling points in (*b*).

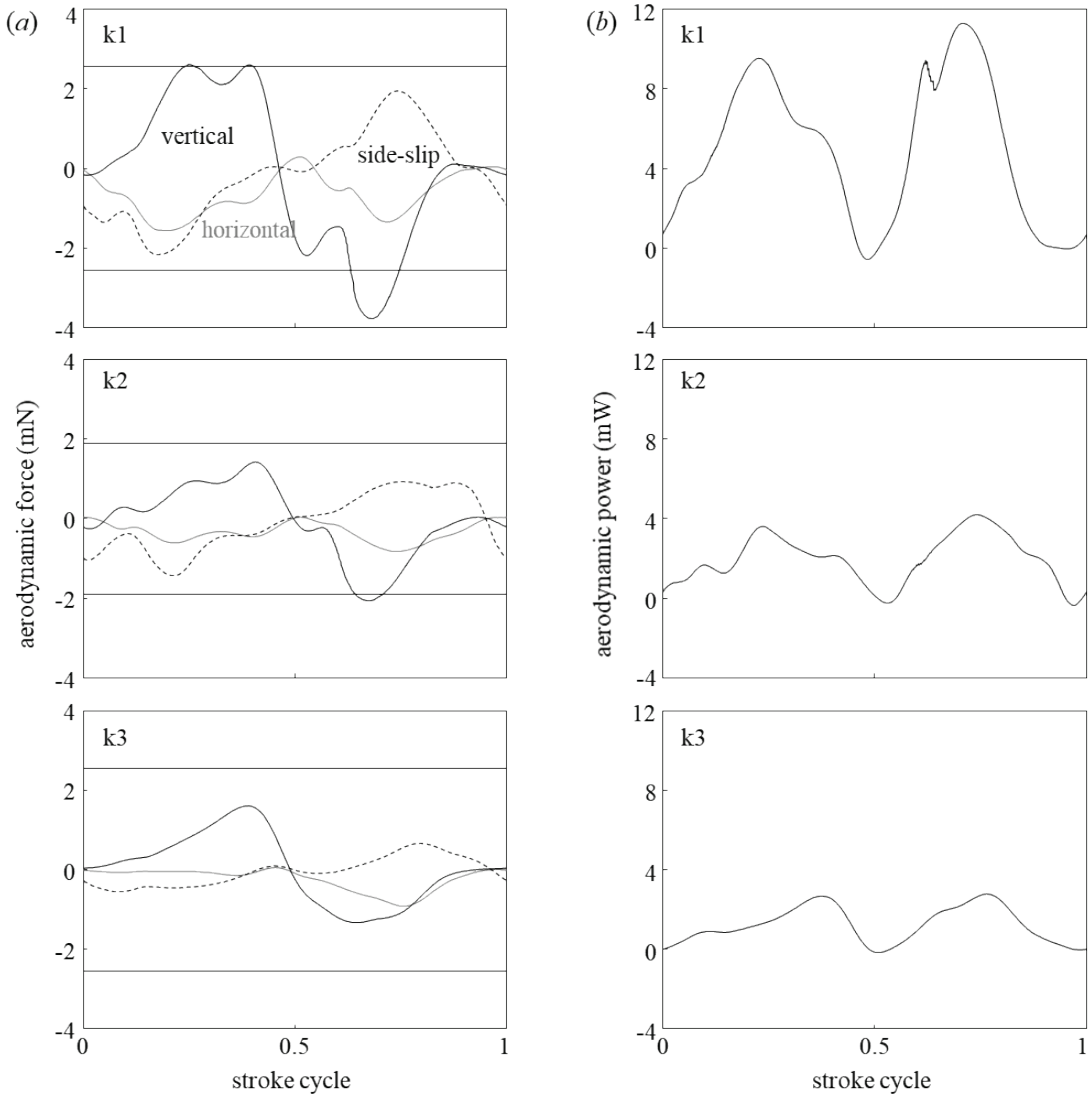

Supplementary Figure 5 Time series of (*a*) aerodynamic force and (*b*) power for each individual (k1-k3). The weights of each individual are shown by horizontal lines for comparison. Horizontal, side-slip, and vertical forces represent the aerodynamic force along the *x*, *y* and *z* axes in Figure 1, respectively. The side-slip force is cancelled when the left and right wings are combined; therefore, the side-slip force for one wing is shown in the figure.

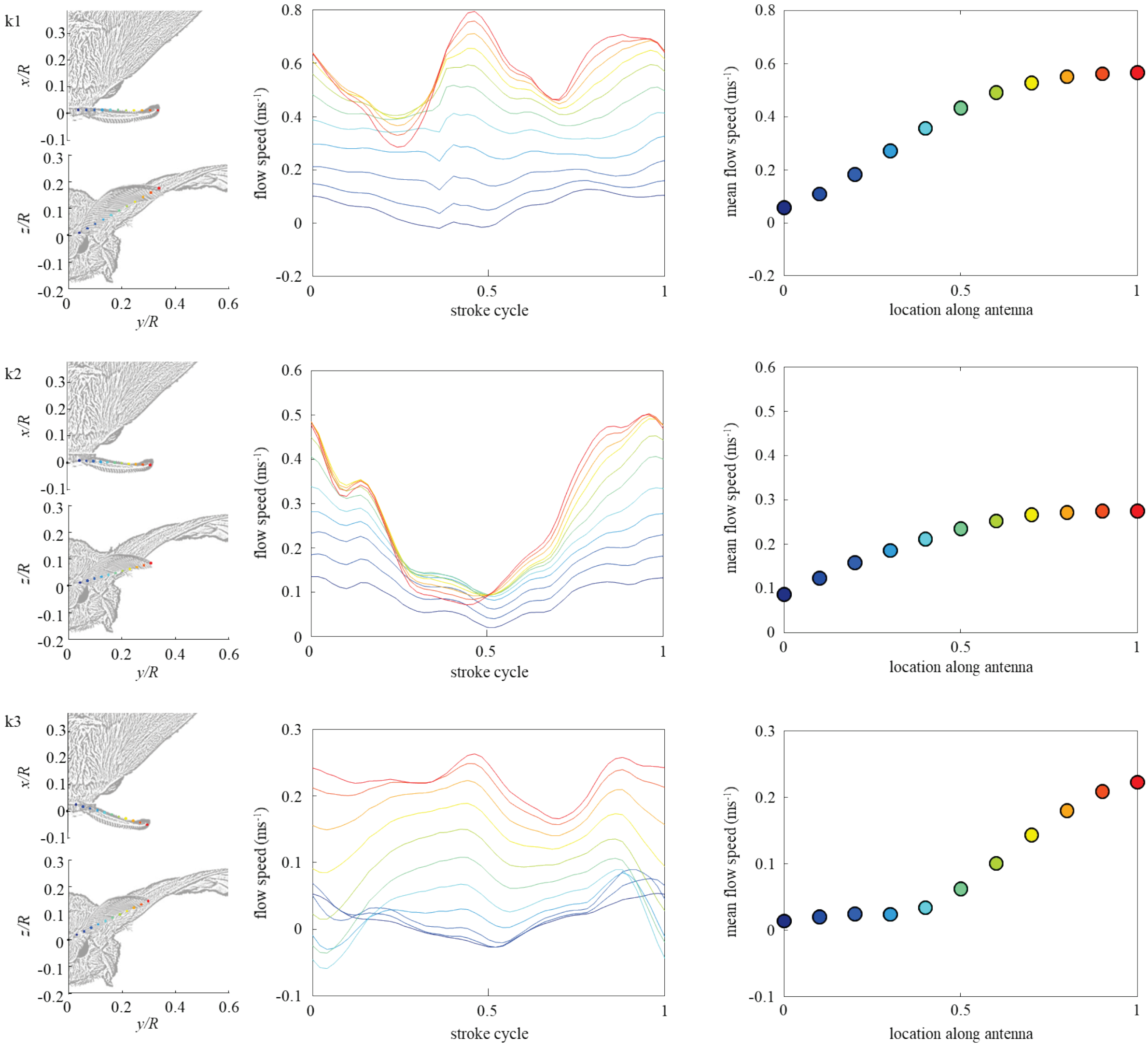

Supplementary Figure 6 (left) Antenna position, (middle) time series of the simulated flow speed at the antenna location, and (right) cycle averaged flow speed from the three individuals (k1–k3).

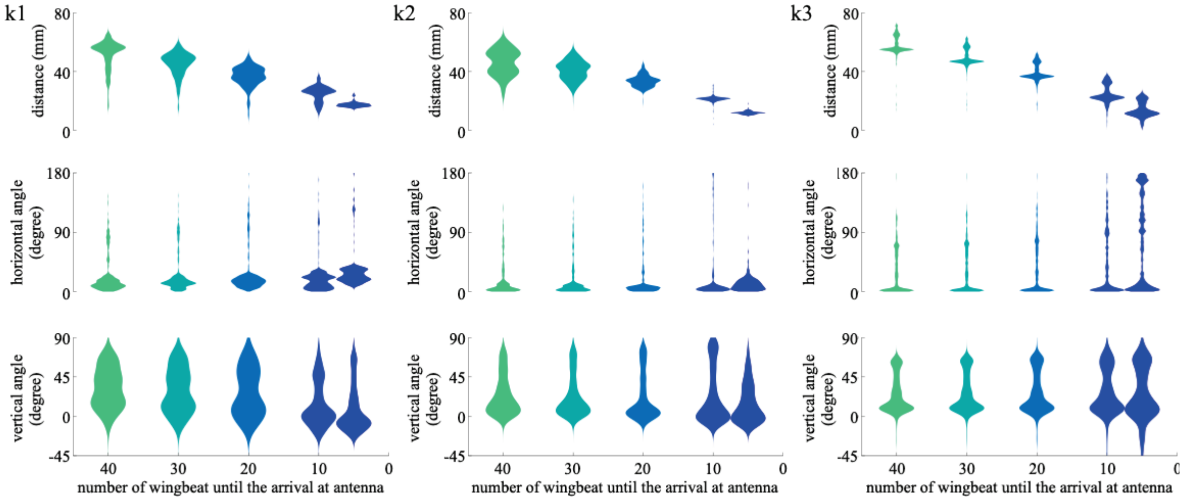

Supplementary Figure 7 Time series of the violin plot of the distance (top), horizontal (middle), and vertical angles (bottom) of the particles with respect to the middle of the antenna. The arrival time of the particles at the antenna is set to zero.

Supplementary Table 1 Wing length and mean wingtip speed of each individual.

individual	wing length, R (mm)	mean wingtip speed, U_{ref} (m s^{-1})
k1	17.7	3.79
k2	17.5	2.70
k3	16.6	2.42
f1	23.9	4.23
f2	24.9	4.34
f3	22.5	3.69
f4	24.2	2.71

Supplementary Table 2 Number of nodes and time steps for verification.

	coarse	finer	finest
Wing grid	41×81×31	51×101×41	61×121×51
Body grid	33×65×15	41×81×21	53×97×31
Cartesian grid	168×84×91	201×101×109	251×126×136
Non-dimensional time step	0.012 (2.58×10^{-5} s)	0.01 (2.25×10^{-5} s)	0.008 (1.73×10^{-5} s)
